# Inclusive recycling movements: a green deep democracy from below

**DOI:** 10.1177/0956247820967621

**Published:** 2020-10-21

**Authors:** María José zapata Campos, Sebastián Carenzo, Jaan-Henrik Kain, Michael Oloko, Jessica Pérez Reynosa, Patrik Zapata

**Keywords:** citizenship, enumeration, grassroots governmentality, grassroots networks, waste pickers’, movement

## Abstract

This paper examines the multiple strategies articulated by grassroots recycler networks to bring about socioenvironmental change. The paper shows how these networks are an emblematic case of grassroots governmentality, whereby urban poor communities contribute to building more inclusive environmental regimes by developing technologies of power more typical of the powerful. These technologies include enumeration, with its resulting self-knowledge; the production of discourses and rationalities of social inclusion and environmental sustainability; and engagement in open and diverse alliances, at times with actors holding apparently antagonistic interests. The paper also reveals how recycling networks are a representative case of deep and green democracy. It is deep democracy, as grassroots networks strive to gain deep and true representativeness in their territories. It is green democracy, as it illustrates alternative pathways to environmental governance that is not limited to state and global organizations, but that also includes a range of control techniques emanating from the communities themselves.

**Figure:**
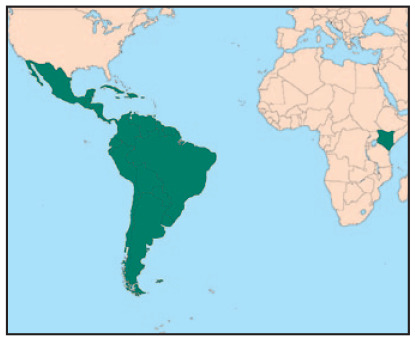


## I. Introduction

Waste pickers are increasingly recognized for their significant contributions to reducing the carbon footprint of cities,^(1)^ recovering resources,^(2)^ improving the environmental conditions and health of low-income residents,^(3)^ and creating jobs and income for the poor.^(4)^ Despite the stigma associated with their work, as well as the low incomes and the poor conditions in which they work, waste pickers tend to be tremendously resilient and well organized, increasingly connecting with each other through city, regional and global waste picker organizations (WPOs) and networks worldwide.^(5)^

Unlike many platforms created by external actors, these bottom-up networks are highly flexible, and their learning by doing and peer-to-peer interacting allow them to navigate contested environments.^(6)^ Collective learning processes have helped these communities create a common voice in engaging with international agencies and industries^(7)^ – resisting, for example, the introduction of incineration technologies.

Urban social movements can follow multiple strategies to prompt change.^(8)^ Unlike social movements mobilizing open contestation in the streets and squares, urban poor communities of slum dwellers and waste pickers challenge the nature of the state through their everyday practices, such as self-help urbanism and informal recycling services.

These new forms of urban social movements have emerged in recent decades in ways that profoundly redefine citizenship.^(9)^ Appadurai^(10)^ has argued that these movements are expressions of “deep democracy” constructed through governmentality from below, informed by techniques, such as enumeration and the resulting self-knowledge, and developed by the urban poor rather than the state or international agencies. Still, the concrete contribution of grassroots networks emerging from informal settlement dwellers’ struggles – as representatives of broader movements of insurgent citizenship, and pathways towards more inclusive and livable cities – is under-researched. Based on the concepts of deep democracy and grassroots governmentality, this paper examines the multiple strategies that grassroots waste picker networks articulate and employ to bring about environmental, social and economic change. By studying the case of recycling networks in light of the notion of grassroots governmentality,^(11)^ this paper contributes to expanding the emergent literature on urban social movements.^(12)^ It also develops earlier strands of research on social movements, the city and the grassroots.^(13)^

Our study illustrates how inclusive recycling networks are emblematic of grassroots governmentality whereby urban poor communities help build more inclusive environmental regimes. The paper also reveals how recycling networks are representative of deep, green democracy.

The paper is informed by life stories and interviews with members of three waste picker networks in Africa and Latin America: one local, one national and one regional. It starts with an overview of the inclusive recycling movement (Section II) and a presentation of the theoretical framework (Section III), followed by a description of the methods of data collection and analysis (Section IV), and exploration of the three networks studied (Section V). The paper ends with a discussion of the findings (Section VI) and conclusions (Section VII).

## II. The Inclusive Recycling Movement

WPOs adopt diverse organizational forms in different parts of the world. In Latin American countries, waste pickers are often organized into cooperatives and associations.^(14)^ This follows and redefines the historical pathway of cooperativism in this region,^(15)^ which was supported in the 2000s by progressive public policies.^(16)^ In East African countries, WPOs often take the form of self-help groups and community-based organizations (CBOs) or comprise small entrepreneurs and associations of entrepreneurs. Some of these are embedded in existing institutional arrangements for the self-organization of civil society in informal settlements.^(17)^ Regardless of their organizational form, they all tend to develop interorganizational networks in order to facilitate material acquisition, the negotiation of prices, government advocacy or knowledge exchange.^(18)^

Still, there are significant differences between WPOs in different regions. In East Africa, interorganizational networks are still few and younger.^(19)^ In Latin America, WPOs are often organized within national-scale networks, and in countries with a longer tradition of self-organization, such as Brazil or Argentina, they can have huge memberships. At the regional level, the Latin American Recyclers Network (RedLacre) comprises national movements from 17 countries and represents more than 1.2 million recyclers. The Global Alliance of Waste Pickers brings together thousands of WPOs from over 28 countries, covering mainly Latin America, Asia and Africa.^(20)^

These overarching networks have helped strengthen the role of local networks and WPOs, for example, by directing public policies towards inclusive recycling models, increasing access to specific funding, and supporting skills training.^(21)^ In terms of environmental policymaking, networks, such as RedLacre have succeeded in participating in climate change summits, strengthening the global anti-incineration movement, and lobbying for more sustainable waste management policy agendas.^(22)^

Nevertheless, these networks are often difficult to sustain due to the scarcity of resources and the multiplicity of interests involved. Conflicts on issues of representativeness, internal governance and transparency are also challenging.^(23)^ Some networks can become unstable, while others disappear or become dormant.^(24)^ Given the relevance of grassroots networks, their contribution to sustainability and their interlinked strategies to prompt socioenvironmental change merit further research.

## III. Grassroots Governmentality and Deep Democracy

The notion of governmentality facilitates an understanding of power and governance that goes beyond state politics to embrace an extensive set of technologies for social control, including the active participation of individuals in their own governance and self-disciplining.^(25)^ For populations and spaces to be governed, they must be identified, classified and ordered through technologies, such as enumeration and statistics. More recent scholarship in governmentality has shown how urban poor communities can realize, in Appadurai’s words, processes of “countergovernmentality” or “governmentality from below” to *“mobilize knowledge about themselves in a manner that can resist eviction, exploitation and surveillance in favour of advancing their own rights, resources and claims”*.^(26)^ Through enumeration, communities are not only empowered by the self-knowledge they develop; in addition, the process of enumeration is performative and facilitates the creation of the community itself.^(27)^ These new forms of organized power and expertise emerging from urban poor communities constitute efforts to redefine citizenship, in what Appadurai^(28)^ has called “deep democracy”. Building on these efforts, Roy^(29)^ developed the concept of “civic” or “grassroots” governmentality to overcome the distinction between governmentality from above and below. Instead, Roy argues that forms of grassroots governmentality *“both resist and comply with what may be perceived to be top-down forms of rule, be it those emanating from the state or from international institutions”*.^(30)^ In a grassroots governmentality regime, the community is therefore both empowered and self-disciplined.

Grassroots governmentality is rooted in the community’s engagement with the politics of knowledge through everyday practices – for example, in processes of self-help urbanism or the self-provision of critical services, such as community toilets and informal waste collection. It is therefore intimately connected to the insurrection of subjugated knowledges developed by the urban poor who, in Foucault’s words, *“have been disqualified as inadequate to their task”*.^(31)^ In the context of this paper, for example, this would include the socioenvironmental competencies and technological innovations developed by waste pickers through their daily work. Such knowledge, developed within the informal city and the informal economy, challenges the ideal of modernity and rationality that city planners and politicians envision in their plans.^(32)^

These a priori unruly territories (e.g. dumpsites and informal settlements) and practices (e.g. waste picking) remain illegible and unfamiliar to outsiders, such as the state. However, they are not illegible to insiders, who can develop relations of either solidarity or exploitation – for example, through patronage, brokerage or political clientelism – with powerful local actors, such as landlords or what de Wit and Berner call community “grasstops”.^(33)^ On the one hand, this external illegibility has led to longstanding marginalization and stigmatization, hindering these communities from accessing basic citizenship rights that, to some extent, are guaranteed by governmental institutions.^(34)^ On the other hand, this unintelligibility has historically contributed to a certain degree of isolation and autonomy from external interventions.^(35)^ The state, city planners, corporations and international agencies aiming to address poverty, employment, housing and waste management strive to overcome this unintelligibility and make these territories and practices transparent. Subjects and spaces must be made legible to be governable. This is achieved through techniques by which the state exerts power, for instance through its enumeration of citizens, gathering of statistics, and programmes for formalizing informal economies and infrastructures^(36)^, such as bulldozing informal settlements.^(37)^ These state techniques also include the introduction of modernizing technologies at dump sites,^(38)^ which often result in the privatization of waste management services rather than the improvement of waste pickers’ working conditions.

## IV. Methods

This paper is informed by three case studies of recycling networks operating at different levels: citywide (the Kisumu Waste Actors Network [Kiwan] in Kisumu, Kenya), national (the Nicaraguan Waste Picker Network [RedNica]), and regional (RedLacre in Latin America). One selection requirement was that the network in question be operational and stable, as some recycling networks exist more on paper than in practice. Another requirement was that we, the researchers, be familiar with the network. We have studied the selected networks, in two cases since their inception, giving us privileged access to data. RedLacre, a network that spans a continent, is unique, as there is no other regional grassroots network of this kind. RedNica is a national network of waste picker cooperatives rooted, like other Latin American national recycling organizations, in the cooperative movement. Kiwan represents a nascent but very active local network that unites a diverse array of groups typical of East African countries, including CBOs, women’s groups and youth groups together with micro and small entrepreneurs.

The cases were built through a combination of network members’ life stories and interviews, meeting observations, site visits, desk research, and study of media documents. A total of 49 in-depth interviews (15 in Kiwan, 12 in RedNica and 22 in RedLacre) were conducted between 2017 and 2019 with participants selected for their diversity in terms of gender and status (Table 1).

**Table 1:** Recycling networks included in the study

	Year of creation	Level	Geographical scope	Members	Funding
Kiwan	2017	City network	Kisumu, Kenya	41 waste picker organizations	Member fees
RedNica	2008	National network	Nicaragua	9 cooperatives, 3,000 waste pickers	Member fees, projects and sponsors
RedLacre	2005	Regional network	Latin America and Caribbean	17 national networks, 1,200,000 waste pickers	Projects and sponsors

Our research began inductively with collecting and coding the empirical data. As we got to know the cases better, data coding in a first-order analysis^(39)^ revealed the emergence of multiple categories, e.g. mapping, recruitment and knowledge. We then grouped these categories into three emerging second-order aggregate themes – enumeration and knowledge, rationalities and identity, and relationships – that recalled theoretical concepts from the scholarship of governmentality, specifically grassroots governmentality. We continued by simultaneously completing our data collection and refining the analysis,^(40)^ but now with a particular focus on the three main themes.

The preliminary findings stemming from the analysis were discussed with members of the networks in several meetings organized in Kenya, Tanzania and Chile during 2018 and 2019. These discussions have served to add nuance to our results and increase the validity of our findings.

In the following section, we present the findings from the three case studies, following the coding from the inductive analysis of the interviews in the first-order analysis.

## V. Recycling Networks

### a. Kiwan

Kiwan is a cooperative society of micro and small waste entrepreneurs, CBOs, and youth and women’s groups in Kisumu, Kenya, founded in 2017 to advocate for improved working conditions for waste pickers.

#### Mapping, recruitment and legitimacy

An important initial task for all members is to enrol new participants. This not only serves to contribute to Kiwan’s representativeness in the eyes of local government, but has also led to the mapping of new WPOs, constructing a more broad-based and committed membership. For example, youth groups in the Obunga informal settlement enrolled women’s groups working with fish waste, framing them as WPOs for the first time.

Kiwan is also recognized for its members’ accumulated knowledge and resources, as workers live alongside other residents in the informal settlements where the city has no capacity to provide waste collection services. One Kiwan member stated:,

“We as local actors know how to interact with residents. . .better than does the county government. When a resident sees a county officer they start running [i.e. because of fear of sanctions], they know the officer will come with reinforcements. . .The county has realized that they have to go through the network.” (Interview D)

Kiwan’s activities are embedded in relations of friendship and kinship in the informal settlements, and their familiarity with these settlements (and hence the legibility of the settlements) becomes an asset when Kiwan appears before the local government.

#### Identity and stigma

For the most marginalized members – e.g. the youth working at the dumpsite – participation in Kiwan has been transformative. Working with waste has become a source of pride rather than stigma:,

“Kiwan has helped us to be identified. . .we are recognized by the county. . .Most people fear us street boys – people think we are going to rob them – it’s good if somebody understands you.” (Interview I)

Waste picker groups have also reshaped their role and started monitoring and sensitizing residents about their environmental behaviour. Youth group members, to their own surprise, have started to adopt roles that conventionally fall to the government:,

“We clean up all the places, so people think we are the county – ‘Don’t put that waste there!’ – but we are from here!” (Interview I)

#### Learning

Kiwan members also share business ideas and innovations, exchange customers to increase the efficiency of their service delivery, and identify complementary services and materials to buy and sell from each other:,

“We used to burn polyethylene, but some women make baskets out of it. So now when we pick it, we sell it to these women’s groups.” (Interview K)

Senior members share knowledge with junior groups regarding, for example, issues of safety:,

“I have learned to work more safely – hazardous waste, broken bottles. . .as scavengers we need gloves and boots.” (Interview K)

Kiwan also enables the development of managerial knowledge, e.g. of finance, associational life and even governance:,

“This is my first table banking experience in Kiwan. I am learning so that I can educate my fellows. . .I am also learning how the Sacco [i.e., a savings and credit cooperative] works, as I would like to have a management position in the future.” (Interview I)

Financial and governance literacy remains one of the most important gains for Kiwan members.

#### Inclusion versus exclusion

Kiwan consists of a combination of heterogenous groups, many operating in informal settlements, although some provide services to formal businesses or engage in the processing and resale of recyclables. Still, equity is embedded in the network:,

“We have signed an MOU stating that whether you are big or small, we assume we are all equal. Women are represented equally, older people and young people” (Interview H).

Women and youth members of small groups who were interviewed felt that their voices were heard.

As a network, Kiwan also helps ideas from the members attending meetings to trickle down to their respective groups. The heterogeneity of this network helps connect the grassroots of the most vulnerable communities with other Kiwan members, and with the city, county and beyond – for example, through collaboration with local and international universities and development programmes.

However, these groups do not have equal representation. Men representing more powerful groups, for instance small entrepreneurs, predominate in executive positions. Similarly, established mechanisms linked to meeting attendance, such as the requirement to buy shares, discourage the attendance of less powerful groups, such as the youths working at the dumpsite.

### b. RedNica

RedNica was founded in 2008 as a result of the protests held by waste pickers working at La Chureca, Managua’s municipal dumpsite. These protests were aimed at stopping municipal workers from sorting out recyclables during their waste collection services, which left waste pickers without income. Since then, RedNica has worked to improve waste pickers’ labour rights and to advocate for waste management policies in Nicaragua that support more sustainable and inclusive recycling.^(41)^

#### Recruitment and network construction

Members of RedNica comprise both WPOs and individual waste pickers. RedNica claims to represent 3,000 of the more than 13,000 waste pickers in the country. Nine WPOs are affiliated with the network, most of them founded by RedNica. It was easier to integrate new groups than to negotiate with and recruit from the few that already existed.

In 2014, a study to identify and evaluate waste pickers’ working conditions at landfills was conducted by RedNica with support from the Central American University (UCA) and WIEGO, the global network of informal workers. This and other enumeration efforts were used to survey individual waste pickers and to form new member WPOs. The results of this study revealed for the first time the contribution of waste pickers to the country’s waste management systems:,

“[We agreed on] the need to produce a document that could be used as a platform to render visible the living and working conditions of waste picker communities throughout the country, and that could serve as a basis for public decision making with regard to this population, so that public waste management policies would integrate their real needs.”^(42)^

#### Enrolling partners

RedNica quickly established relations with a wide array of organizations (including the National Recycling Forum, Fonare and Fundación Avina) to mobilize resources and legal support. In 2010, RedNica was invited, through Fonare, to participate in the fourth Latin American Congress of Waste Pickers hosted by RedLacre. A year later, the Nicaraguan network was invited to act as the operational secretariat of RedLacre for the 2012–2014 period. Over this period, RedNica organized a census in Central America to identify and form national recycling movements in Panama, Costa Rica, Honduras, El Salvador and Guatemala.

#### Knowledge, advocacy and environmental activism

Participation in regional networks facilitated knowledge sharing about various issues, including the threat of incineration technologies and the privatization of waste management services in other Latin American countries. In 2015 in El Salvador, RedNica, together with WPOs from other Central American countries, protested the privatization of municipal landfills and the introduction of incineration technologies. The anti-incineration narrative has been deeply integrated into the national movement since then. Similarly, contact with other RedLacre members supported the development of an interest in environmental issues beyond waste, such as water issues:,

“We contribute to [better] health, we support the environment and. . .even though we are apolitical, we contribute to issues debated in current politics, to waste management policies, to water [policies], which is a theme that is relevant to all citizens regardless of whether one is a *churequero* [i.e. a waste picker working at La Chureca dump] or a professional, it is our responsibility.” (Interview D, RedNica)

RedNica also participated in the development of several waste management laws in Nicaragua that now acknowledge waste pickers as legitimate actors in the waste management system. Recently, however, lack of transparency in RedNica’s accountability to other organizations, insufficient administrative capacity and oversized leadership in Managua have led to a loss of membership, increasing conflict, and worsening relations with Fonare, Jóvenes Ambientalistas and RedLacre. These conflicts culminated in the head of RedNica terminating the network’s membership in RedLacre in 2018. Current state oppression with serious human rights violations, the subsequent economic crisis with companies filing for bankruptcy, and declining resources from external organizations have accentuated the crisis for the inclusive recycling movement in Nicaragua, leading to the closure of several cooperatives.

### c. RedLacre

RedLA (Red Latinoamericana de Recicladores – Latin American Network of Waste Pickers) was created in 2005 with national recycling movements from Brazil, Colombia, Argentina and Uruguay. In 2011, the organization was renamed RedLACRE (Red Latinoamericana y del Caribe de Recicladores – Latin American and Caribbean Recycling Network of Waste Pickers) to reflect the membership of national recycling movements from Caribbean as well as Latin American countries, 17 in all. There are no membership fees and responsibilities are undertaken on a voluntary basis, unless temporary project funding is available to help sustain member activities. RedLacre is organized around three secretariats (communication, international agenda, and operations and projects), which are assigned to different countries.

#### Mapping and enumeration

Mapping and enumeration have been used by RedLacre since its inception, for several reasons. First, they serve to “expand frontiers” (Interview S) and support the formation of new groups in other countries. In practice, mapping and enumeration cannot be carried out by anyone who is not a waste picker, as they involve visiting isolated and often insecure places, such as dumpsites and informal settlements:,

“We did not contact RedLacre ourselves. It was the *compañero* from Nicaragua who had the mission of recruiting waste pickers [from Central America]. . .When he arrived in El Salvador, I don’t know how he managed – he surprised me when he contacted me.” (Interview Am)

Second, mapping and enumeration serve to verify the authenticity of the waste picker groups – *“knowing that they are real and not fantasy”* (Interview E). This, again, is something that can only be done by other waste pickers who share the same occupation and speak “the same language”:,

“We observed in 2005 at the meeting in Porto Alegre that many technicians came to the meeting representing countries. Based on one’s own experience, one detects, without a doubt, who is a recycler and who is dressing as a recycler. Because if you put together two engineers, between them, how are they not going to know if they are engineers or not?” (Interview E)

In Latin America, community groups are sometimes created with external support to provide waste collection services. Many become and remain inoperative after funding dries up, or are simply never activated. Similarly, a growing number of organizations have claimed to “represent” the recycling movement, thereby gaining legitimacy, and taking advantage of their intermediary role. Discerning who is a true waste picker, and which groups are real and operational, thus remains crucial to RedLacre, indicating an ambition to create a genuine network. This ambition also applies to RedLacre’s expanding geographic range to include the Caribbean and Central America. This is why the mapping in Central America started with a project assigned to RedNica, the only member of RedLacre in that region at that time. This determination to achieve territorial representativeness also applies within each country, where the deep representation of groups beyond the capitals is sought.

Third, mapping efforts have been used to produce scientific data about the network’s environmental impacts, in order that waste pickers can be included in evidence-based policy formulation, as has succeeded in Panamá and Costa Rica:,

“[The idea of the enumeration] is therefore to dispose of facts in such a way that the recycling groups in the countries, when they are participating in decisions regarding a law, or when there are discussions of municipal directives or programmes, the recycling leaders participate properly and do not just receive pity. No! ‘Here we have the data that tell you, you, stop the nonsense because we have an impact. We have challenges but we also have an impact.’ Otherwise, if we had not conducted an enumeration, for example, in Panamá, there would not be a basis for discussion because then they would argue, ‘But you are just a group of poor people living on what others throw away.’ Yes, but this group of poor people, we number around 2,000. . . [each collecting] an average of three to four tonnes [of waste] monthly, multiply that for CO_2_ emissions, multiply that, please, for savings in landfilling, let’s start adding energy [saved], let’s start adding water. But then it is not you guessing a number when you met a friend, no. . .” (Interview E)

In negotiations with decision makers, the waste pickers can use data from the enumerations to build their arguments and make their claims, changing their position from just being a group of poor people to being experts with exact data.

#### Learning across the network

Members of RedLacre operate in contexts of permanent crisis. Episodes of environmental or social injustice (e.g., threats of incineration, privatization, illicit competition from municipal waste workers gathering materials, state or gang violence) are reported outside the country through RedLacre. When facing these harsh situations, waste pickers feel the support of RedLacre. They know that they are taken care of and not forgotten:,

“To belong to RedLacre is beautiful. They do not forget us. By WhatsApp, by telephone calls, they contact us. . .The network is always attentive to us. . .They do not let go of our hands.” (Interview Ar)

The network also serves as a learning platform where the achievements and innovations developed by the members are shared from one place to another:,

“Learning is what has been most useful. That empowers us because one can see how the *compas* from other countries have stronger operations, that they have evolved, not only as informal collectors.” (Interview Am)

Learning from the experiences of other countries sets a precedent for members to start constructing new practices or to claim their rights from their governments. For example, in countries, such as El Salvador or Honduras, where the political context is not at all favourable for informal waste pickers, the strategy has been to continue learning and to prepare proposals to be presented when the situation changes:,

“RedLacre increased our expectations, gave us other visions so we could continue, because sometimes I feel like I want to join the [migration] caravans. There are days when I do not earn anything. . .With RedLacre I have learned that in some countries [recyclers] have better living and working conditions: Brazil, Chile, with strong cooperatives, with the support of the governments. Therefore, we hope that [our] governments will listen to our petitions.” (Interview Ar)

“Now in El Salvador. . .it was good to wait. Now we understand the issues. We have learned from other countries.” (Interview Am)

#### Openness, partners and supporters

Over the years, RedLacre has developed a network of technical “supporters” who are both “professionals” and “friends” (Interview E). RedLacre has also built important connections with NGOs, private foundations and corporations. For example, RedLacre is a founding member of the Regional Initiative for Inclusive Recycling (IRR), together with the World Bank, Coca-Cola, PepsiCo and Fundación Avina, which is a platform for investment, knowledge and public policy advocacy regarding inclusive recycling in Latin America.

Nevertheless, despite being partners in certain fora where RedLacre can pragmatically exploit opportunities for funding, some of these organizations can be considered “the enemy” in other situations – for example, when large corporations compete for recycling work under the new system of Extended Producer Responsibility (EPR). This is a policy approach in some Latin American countries, whereby producers are responsible for treating or disposing of recyclables under conditions that prevent recycling cooperatives from gaining access to these resources. Furthermore, these alliances are not uniformly supported. Some members of RedLacre and of environmental organizations remain critical:,

“The new fashion of the circular economy. . .[is one of our main challenges], those who have been polluting the planet, those responsible for generating the residues, they now want waste to be collected without a cost.” (Interview S)

#### Environmental contributions and identity formation

RedLacre has participated in six UN climate change summits and in several UN-Habitat conferences. It has also been involved in the anti-incineration movement in collaboration with Gaia, giving this global environmental movement a deep connection to the grassroots (Interview Gaia). Similarly, the network’s agenda in each country is shaped by, and shapes, other organizations’ agendas, resulting in members supporting diverse protests – for instance against incineration, a cement factory, water privatization or climate change:,

“There were some politicians who wanted to privatize the water services, and we heard about that and we mobilized protests the next day. Someone called me, ‘Do you think you can join us at the demonstration tomorrow?’ The demonstration was called by the National University. The protests against the cement factory were called by an NGO working on zero waste.” (Interview A)

The involvement of RedLacre members in several environmental struggles reflects a shift in their identity from being members of an economic solidarity movement only to also being part of an environmental movement:,

“In everything we do as recyclers, the environment is involved. The environmentalists are not those who work for the environment [in their discourses], it is we, the recyclers! The network is environmentalist. We are the environmentalists! If it was not for us, the recyclers, it would not be possible to save the planet.” (Interview A)

This member of RedLacre reconfigures the identity of the waste pickers and the recycling movement as environmentalist because of their actions (recycling), not based on rhetoric, as is the case with many other environmental organizations.

## VI. Discussion

Informed by the cases of Kiwan, RedNica and RedLacre, this section discusses how these grassroots networks have advanced strategies to promote socioenvironmental change. They have done so by using technologies of enumeration and the resulting self-knowledge, by blending rationalities of economic inclusion and environmental justice, and by mobilizing alliances characterized by their openness and diversity.

### a. Building membership, enumeration and self-knowledge

All three networks devote many of their scarce resources to self-enumeration, constantly scanning and enrolling new members, being aware that representativeness through a broad membership is an important source of legitimacy and power.^(43)^ Waste picker networks can grow either by integrating existing WPOs or by actively creating them, as other meta-organizations often do.^(44)^ RedNica is a good example, as it grew first by mapping and then by enrolling individual waste pickers throughout the country into newly formed WPOs. Yet it failed to integrate existing groups, which affected its long-term internal and external legitimacy. In contrast, Kiwan has managed to pursue a two-tiered approach by both enrolling existing WPOs and supporting the organization of disenfranchised waste pickers into new WPOs. RedLacre, while successfully engaging most of the previously existing national movements, has also contributed to the formation of new national networks in several Latin American countries.

Although building legitimacy and membership is critical, enumerations, community mappings and surveys accomplish much more than just that. These practices of self-research^(45)^ help generate another, even more vital, resource: self-knowledge. By identifying individuals and groups of waste pickers, by characterizing them and measuring their environmental impacts, the networks generate fundamental knowledge in a field about which even local governments lack systematic data. This self-knowledge, first of all, awakens consciousness of the environmental contributions of the waste pickers’ work, as is further discussed below. Second, this self-knowledge is used as scientific evidence when negotiating environmental and waste management policies, using the technologies of power that are generally more typical of the powerful.

Such self-knowledge is both empowering^(46)^ and insurgent.^(47)^ It is empowering since, as Appadurai explains, it *“takes this power away from external agencies such as the state and puts it back to where it truly belongs, which is within the community itself”*.^(48)^ It shifts the centre of expertise from the state and large corporations to urban poor communities. It is also insurgent, as it emanates from the informal settlements and the informal economy where the urban poor constitute, in Holston’s words, *“a more autonomous sphere of self-interested and competent citizens”*.^(49)^

The ability to research or “see” within these communities through enumeration and mapping cannot be taken for granted. Simone^(50)^ has noted the difficulty the untrained eye has in grasping the complex organizational life of African cities, for instance, leading to assumptions that they are “incomplete”. Frequently, it is also nearly impossible for outsiders to access these seemingly unruly territories. The consequence is that the associational life and the contributions of WPOs can be illegible, invisible or seemingly “incomplete” to the eyes of public officers, development aid organizations, and researchers like us. Yet, for residents of informal settlements or for waste pickers working at a dumpsite, the territory and community are familiar and legible. The example of the dozens of women’s groups transforming fish waste into various products in Kisumu’s informal settlements, recruited into Kiwan by a young member and local resident, illustrates how being an integral part of the community gives grassroots networks the ability to “see” sometimes loosely organized and almost invisible groups. This “gaze”^(51)^ – or “grassroots gaze” as we call it – stems from and is shaped by the condition of the community members as waste pickers and slum dwellers. The grassroots gaze is, however, not conferred automatically on community members; rather, the gaze must be refined and enhanced, for example through enumeration work.

The three cases illustrate waste picker aspirations to create networks with representation deeply embedded in the respective territories: within the city of Kisumu and its informal settlements, outside the dumpsites of the capital city of Managua in Nicaragua, and in all Latin American and Caribbean countries. That is, the waste pickers configured networks intended to be strongly rooted in deep democracies^(52)^ and to have inclusive memberships. Also, they strove to build networks with members who faithfully belonged to the profession of waste picking. This striving to create true WPOs was not without tensions, which ranged from identifying which groups were not operational or not truly responding to grassroots movements (i.e., not being supportive organizations), to identifying irregularities and corruption in the democratic processes of network members. The challenge of “seeing” and verifying members’ adherence to the values of democracy and transparency increased as the networks grew, as illustrated when RedNica’s representative ended their membership in RedLacre.

### b. Forging identities through economic and environmental rationalities

Members of the three recycling networks experienced a transformation from a sense of stigma associated with being poor and working with waste, to pride in providing a critical environmental service and being part of a movement for economic inclusion. Their participation in the grassroots networks and the associated enumeration work has transformed their identity. It revealed to them both their economic and environmental contributions, and then reframed the stigmatizing activity of waste picking as a service to society and the environment and as dignified work for the urban poor.^(53)^ By undergoing this “ontological insurrection,” to use Samson’s term,^(54)^ waste pickers have reimagined their own role as active citizens in environmental sustainability governance,^(55)^ helping forge an “inclusive recycling” grassroots governmentality. In this grassroots regime, the waste pickers become both self-empowered and recognized by the state and other actors in their new role as environmental stewards, simultaneously becoming self-controlled and governable.^(56)^ This inclusive recycling grassroots governmentality resonates with what Fredericks^(57)^ has called “garbage citizenship” in Senegal. Through “garbage citizenship”, Dakar’s residents exploit waste infrastructure as a governance technology for forging collective identities and mobilizing political action, and waste can become a technology of governmentality for both control and contestation.

The waste pickers’ identities have also evolved in terms of their economic and environmental motivations. While the struggles for labour rights, economic inclusion and recognition represented their initial aspirations, they have gradually incorporated an environmental rationality into their discourses. This rationality was pre-existing in their waste collection and recycling practices, but not explicitly elaborated on in their discourses. Regardless of the sequence, economic and environmental rationalities are intertwined.^(58)^ Rather than being static or fixed, the members’ rationalities are situational and fluid: depending on the context, their identities oscillate between that of activists in a labour movement striving for the economic inclusion of the urban poor, and that of environmental stewards. Network members adhere to fluid identities^(59)^ drawing pragmatically on a repertoire of ambiguous rationalities. In Foucault’s words, these ambiguous rationalities serve to *“found, justify and provide reasons and principles”*^(60)^ for their actions, and enable them to access further resources in resource-poor, complex, and often coercive contexts.

### c. Relations based on openness and diversity

Contestation, often in reaction to processes of displacement and eviction driven by local governments and other state agencies, is a typical strategy articulated by all three networks since their inception. But collaboration through numerous alliances is also a common strategy. For example, although the foundation of RedNica was triggered by the protests of waste pickers at the La Chureca dumpsite, its development was as much the result of cooperation with environmental organizations and other supportive actors, such as RedLacre. Also, RedLacre and its constituent national movements created strong ties with social movements and left-wing political coalitions in the territories where they emerged.^(61)^

Many alliances also pragmatically include heterogeneous actors, such as state agencies and large corporations. That is, recycling networks are characterized by the openness and diversity of their relationships with powerful actors, including partnerships more typical of neoliberal governmentality.

One implication of the openness and diversity of this grassroots governmentality is the enrolment of apparently opposed actors in collaborative alliances that resemble what Gibson-Graham et al.^(62)^ have called “multi-species communities”, in which organizations with antagonistic interests converge temporarily. This feature is shared by other new environmental network movements.^(63)^ One example is provided by RedNica. While some cooperative members were collaborating with the Nicaraguan Trade and Cooperatives Ministry to sell their recycled products at a trade fair for social entrepreneurs, the materials used to produce these items were collected illegally from the municipal landfill, where the police were persecuting the same waste pickers who participated in the trade fair. As Mitlin^(64)^ has demonstrated, urban social movements pragmatically draw on multiple strategies, ranging from collaboration to silent resistance and open contestation, to secure their aims.

RedLacre also illustrates this strategy of openness to heterogeneous actors, for example through its participation in the IRR, in which actors, such as Coca-Cola and the World Bank are founding members and sponsors. The support of some IRR activities has strengthened RedLacre’s advocacy work with several national governments, resulting in the development of more inclusive national waste management policies and legal frameworks. RedLacre members are nevertheless aware of the risks of this collaboration between “strange bedfellows”. While some members have reservations about some of the funds coming with strings attached, others simply distrust large corporations. Through its participation in the IRR, Coca-Cola, *“one of the largest polluters of the planet”* – in the words of one interviewee – is suspected of greenwashing. Also distrusted is the future role of these large corporations under the new Extended Producer Responsibility (EPR) policy approaches in many countries. Despite these tensions, RedLacre has pragmatically drawn on technologies of governance typical of the powerful (e.g. knowledge generation through enumeration, mapping, assessment of environmental impacts, awareness campaigns, partnerships and market alliances) to build a grassroots governmentality.

Even coalitions with actors whose principles are apparently closer to those of the recycling networks, such as environmental organizations, are not free of controversy. Some more radical members of Gaia, the global anti-incineration organization, might disagree with RedLacre’s pragmatic strategy of collaboration with actors, such as Coca-Cola, which cause global pollution. Conversely, WPOs would disagree with certain notions of zero waste, arguing that without waste their source of income would be extinguished, and that a circular economy also has to be “inclusive”.^(65)^

As a result of the openness and diversity of their relationships, recycling networks are incessantly engaged in redefining and maintaining the boundaries^(66)^ of inclusive waste picking to preserve their autonomy from attempted co-option^(67)^ by large corporations, development agencies, states and environmental organizations. At the same time they are continuing to work in alliances with supportive organizations, social and environmental movements, governmental actors and large corporations to achieve their goals.

In sum, the openness and diversity of the WPO alliances result in “multi-species communities”^(68)^ that enable resilient grassroots governmentality in resource-scarce contexts. Yet these open alliances generate tensions that are balanced, for example, by “juggling”^(69)^ or conducting multiple activities and relations simultaneously – or in a “meanwhile” time as Carenzo refers to it.^(70)^ The open and diverse alliances the WPOs engage in also have consequences for redefining what “grassroots” are and what “from below” means. Our findings reveal that grassroots networks are configured from different directions, with some external actors moving inwards to mobilize and empower the grassroots, at best facilitating more horizontal knowledge exchange.^(71)^ Simultaneously, through these interactions, grassroots networks also influence supportive actors – as, for example, when RedLacre shaped Greenpeace’s anti-incineration agenda or promoted an approach to the “zero waste” ideal that encompasses the interests of grassroots communities. Consequently, grassroots governmentality implies, first, that the insurgent grassroots both comply with and resist rules and practices stemming from state and international agencies; and, second, that through open strategies of collaboration, the grassroots partake in the “governmentalization” of the state and other market actors by which waste pickers become “insiders” in the enactment of governmental policies.^(72)^

## VII. Conclusions: Grassroots Governmentality, a Green Deep Democracy

Informed by the case of the inclusive recycling movement, we have examined how grassroots networks that emerge from urban poor communities prompt socioenvironmental change. We argue that the result of the colossal and “quixotic”^(73)^ collective efforts of millions of waste pickers is yet another expression of “grassroots governmentality”^(74)^ forging a *green deep democracy*.

### a. Grassroots governmentality

First, inclusive recycling networks are an emblematic case of grassroots governmentality. In grassroots governmentality, communities construct and participate in more inclusive environmental regimes by developing technologies of power typical of the powerful, such as enumeration and the resulting knowledge about themselves, the production of discourses and rationalities, and open and diverse alliances.

This knowledge emerging from the cracks of the formal city, the formal economy and formal waste management systems challenges orthodox scientific knowledge of environmental governance, shifting expertise and power from public officers and experts into the hands of the urban poor.^(75)^ Self-knowledge also empowers,^(76)^ emancipates and transforms waste pickers into more autonomous, competent and “insurgent” citizens.^(77)^ Through the tacit knowledge that emanates from their everyday practices – what Scott^(78)^ has called the infrapolitics of small acts, such as waste collection and recycling – the urban poor can reimagine themselves as citizens with rights, as dignified workers, and as providers of a critical environmental service.

The grassroots governmentality that these networks weave also creates new fields of visibility that shed light both on socioenvironmental injustices and on the contributions of forgotten groups and neglected territories. However, their visibility also conveys their self-disciplining (e.g. adopting the role of environmental educators or formalizing their activities to be included in urban waste management systems, resulting in their fiscal and legal control), and the consequent transformation of waste pickers, informal settlements and dumpsites into more governable subjects and spaces.^(79)^ On the other hand, our findings also illustrate how these networks learn to deal with their self-disciplining – for example, by circumventing bureaucratic requirements^(80)^ and by adopting multiple strategies simultaneously.^(81)^

### b. From grassroots governmentality to deep democracy

Second, recycling networks are also a representative case of deep democracy.^(82)^ Despite the turbulent environments in which they operate, grassroots networks strive to gain true representativeness within their territories, both between groups and within the occupation of waste picking. Their grassroots gaze can penetrate the illegibility of apparently unruly territories. Still, the gaze is not a given, but is instead developed, for example, through technologies of enumeration. Moreover, the search for in-depth territorial and occupational representation has not been without tensions. These tensions are addressed by developing systems of self-scrutiny and control that define the boundaries^(83)^ of the “true” waste picker and avoid co-option by the organizations they collaborate with.

### c. The recycling network movement, a green deep democracy

Finally, we argue that the case of the inclusive recycling movement also represents instances of “green deep democracy”. In the predominant neoliberal governmentality, market mechanisms dominate and state actions are restricted to facilitating market agency and citizen responsibility for environmental change. The green grassroots governmentality studied here illustrates alternative pathways whereby environmental governance is not limited to state and global organizations, but instead includes a range of control techniques emanating from the communities themselves, which simultaneously comply with, resist, and shape state and global corporate politics. Despite emerging from some of the most vulnerable urban collectives, recycling movements have realized considerable achievements in local, national and global environmental governance. They have resisted incineration and the privatization of environmental services, campaigned for policies fighting climate change, and advocated for more sustainable waste management solutions.

This green and inclusive governmentality is based on the waste pickers’ ability to draw pragmatically on a repertoire of ambiguous rationalities of social inclusion and environmental sustainability. Together, these rationalities create and broaden the grounds, principles and resources for their actions. Learning from the emergent role of grassroots waste picker networks has implications for the organization of environmental governance and change, offering new forms of association that are more deeply democratic and green, along with new opportunities to rethink global challenges, such as natural resource depletion and the climate crisis.

## Notes

1. da Silva Carvalho (2012).

2. Navarrete-Hernandez and Navarrete-Hernandez (2018).

3. Dias (2016).

4. Rebehy et al. (2017).

5. Azevedo et al. (2018).

6. Boonyabancha and Mitlin (2012).

7. Mitlin (2015).

8. Mitlin (2018).

9. Holston (2009).

10. Appadurai (2001).

11. Appadurai (2001); Roy (2009).

12. E.g. Mitlin (2018).

13. Castells (1983).

14. Zapata Campos and Zapata (2013).

15. Carenzo (2016).

16. Dias (2016).

17. Azevedo et al. (2018).

18. Dias and Samson (2016); Tirado-Soto and Zamberlan (2013).

19. Gutberlet et al. (2016).

20. GlobalRec (2020).

21. Dias (2016).

22. Fernández (2013).

23. Tirado-Soto and Zamberlan (2013).

24. Zapata Campos et al. (2019).

25. Dean (1999).

26. Appadurai (2012), page 639.

27. Appadurai (2012).

28. Appadurai (2001).

29. Roy (2009).

30. Roy (2009), page 160.

31. Foucault (1980), page 8182.

32. Aparicio and Blaser (2008).

33. de Wit and Berner (2009).

34. Samson (2009).

35. Scott (1998).

36. Carenzo and Fernández Álvarez (2011).

37. Rademacher (2009).

38. Hartmann (2018).

39. Gioia et al. (2012).

40. Charmaz (2016).

41. RedNica (2017).

42. UCA (2014).

43. Deephouse et al. (2017).

44. Ahrne and Brunsson (2011).

45. Appadurai (2012).

46. Patel and Mitlin (2002).

47. Holston (2009).

48. Appadurai (2012), page 640.

49. Holston (2009), page 258.

50. Simone (2004).

51. Foucault (1977).

52. Appadurai (2001).

53. Dias (2016).

54. Samson (2009), page 19.

55. Samson (2015).

56. Roy (2009).

57. Fredericks (2018).

58. Gutberlet et al. (2016).

59. Zapata Campos et al. (2020).

60. Foucault (1991).

61. O’Hare and Sorroche (2019).

62. Gibson-Graham et al. (2016).

63. Goodman et al. (2012); Levkoe and Wakefield (2014); Zapata Campos and Zapata (2017).

64. Mitlin (2018).

65. Azevedo et al. (2018).

66. Lamont and Molnar (2002).

67. Roy (2009).

68. Gibson-Graham et al. (2016).

69. Mitlin (2018).

70. Carenzo (2017).

71. Holt and Littlewood (2017).

72. Roy (2009), page 161.

73. Appadurai (2001), page 42.

74. Roy (2009).

75. Appadurai (2001).

76. Patel and Mitlin (2002).

77. Holston (2009).

78. Scott (1990).

79. Roy (2009).

80. Carenzo and Fernández Álvarez (2011).

81. Mitlin (2018).

82. Appadurai (2001).

83. Lamont and Molnar (2002).
